# Intrauterine Volvulus: A Case Report

**DOI:** 10.7759/cureus.47712

**Published:** 2023-10-26

**Authors:** Tzu-Yao Yang, Wei-Chih Chen

**Affiliations:** 1 Department of Obstetrics, Gynecology and Women’s Health, Taichung Veterans General Hospital, Taichung, TWN

**Keywords:** whirlpool sign, mesenteric defect, intrauterine volvulus, distended bowel, coffee bean sign

## Abstract

Intrauterine volvulus is an extremely rare disorder. Its diagnosis is difficult to make antenatally, unless typical pictures are obtained. Owing to its high morbidity and mortality, intensive monitoring of the fetal condition is mandatory. Here, we report a patient, gravida 4, para 1 (G4P1), who had suffered from preterm labor and diminishing fetal movement and was brought to the emergency unit after her 35th week of pregnancy. Ultrasonography was performed to reveal a fetus with a markedly distended bowel (18 mm in width). Intestinal obstruction related to intrauterine volvulus was highly suspected. The fetus was delivered via Cesarean section because of its deteriorated abdominal condition. Urgent exploratory laparotomy was done by a pediatric surgeon to relieve the bowel obstruction on the second day after birth, on the account of poor improvement of the newborn. The newborn did well after segmental resection and was subsequently followed up at the pediatric outpatient clinic. In conclusion, early detection, intensive monitoring, prompt delivery, and urgent surgical intervention are the key to save the fetal life and neonatal health.

## Introduction

Volvulus, while not rare in young children [1], may often result in high mortality and morbidity. Bowel volvulus is usually caused by bowel loops over twisted, twisting of mesenteric vessels, which leads to congestion or bowel necrosis. Over half of reported cases with fetal midgut volvulus either died in utero or ended with abortion [1], so it is rare and not well studied during the intrauterine life. Its images can be obtained with ultrasonography. Here, we report a case of intrauterine volvulus and discuss its management and prognosis.

## Case presentation

**Figure 1:**
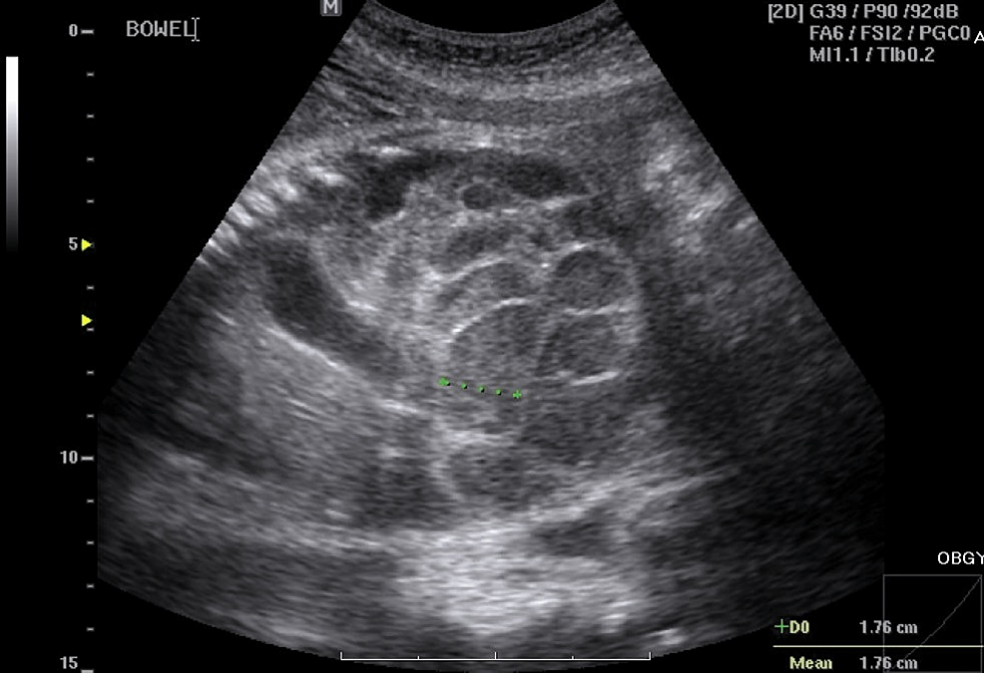
Markedly dilated intestine found by ultrasound

A 39-year-old female, gravida 4, para 1, abortion 2, has a history of epilepsy, which is controlled with carbamazepine and lamotrigine. She also has antiphospholipid syndrome, which is treated with Bokey, Arixtra, and Plaquenil. Antenatal care was unremarkable. However, she visited an emergency unit on her 35th week of pregnancy due to preterm labor signs and diminishing fetal movements. Ultrasound showed a female fetus with a dilated small intestine (18 mm in width) (Fig. 1). Intrauterine volvulus was highly suspected despite no definitive whirlpool sign. However, there was definitely an intestinal obstruction. We kept a close follow-up of her situation with sonography and cardiotocography.

**Figure 2:**
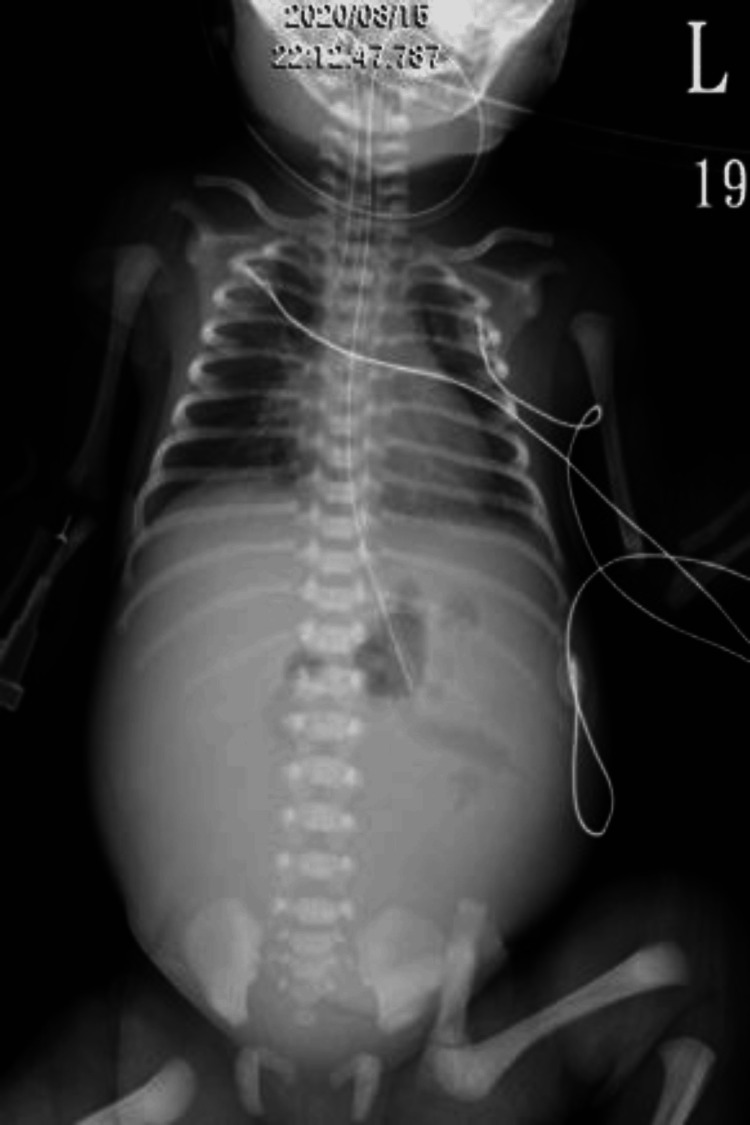
Deteriorated neonatal abdomen

**Figure 3:**
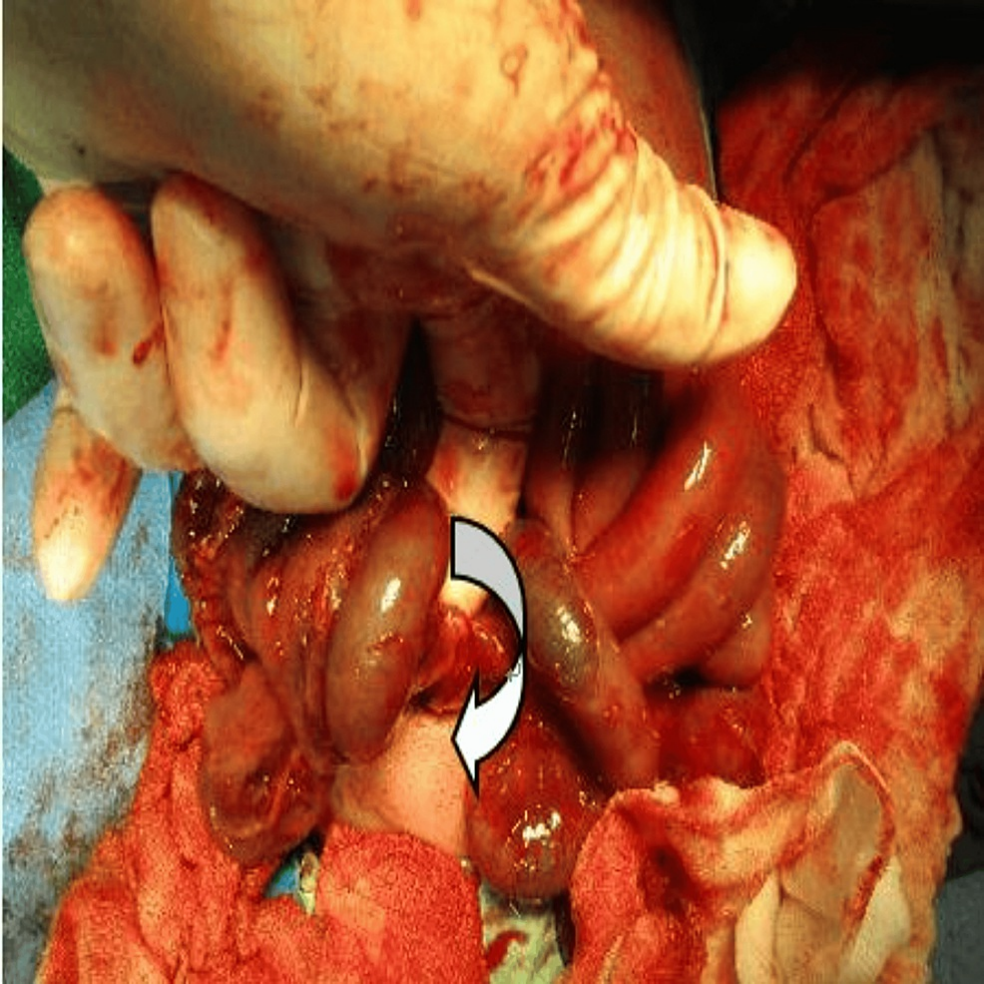
Volvulus resulted from the herniation of the small bowel through a mesenteric defect (arrow)

**Figure 4:**
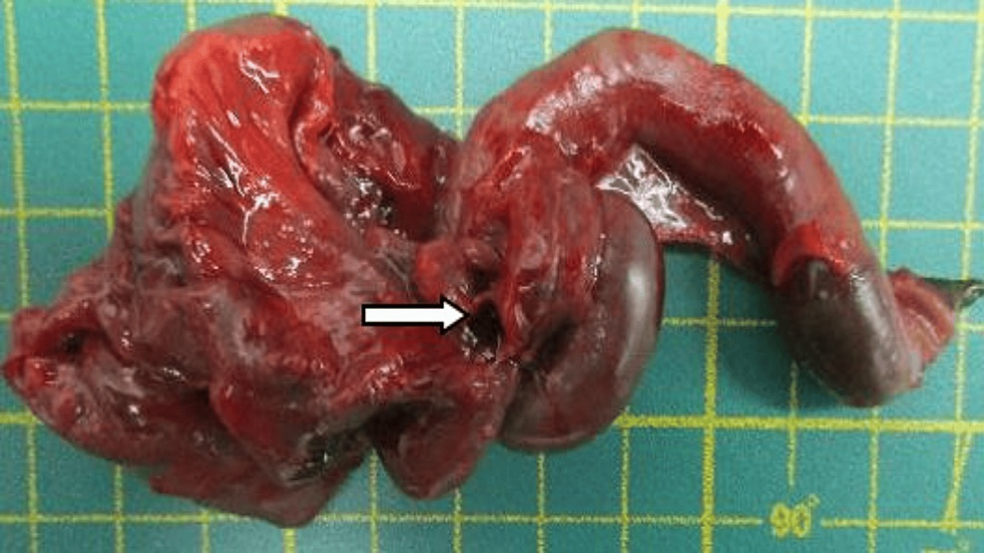
A perforation was found on the resected necrotic bowel (arrow).

She underwent a cesarean section due to fetal distress at the 35th week of pregnancy. A 2,450-gm girl was born with Apgar scores of 3, 5 and 7 at 1st, 5th, and 7th minutes, respectively. Abdominal distension and reddish discoloration were detected after birth before Neopuff bagging, and bowel perforation was suspected. She was sent to the NICU for further evaluation and management. Owing to no improvement in her bowel condition (Fig. 2), segmental resection of small bowel with double-barrel enterostomy was performed the day after birth to reveal volvulus of the small intestine due to internal herniation through a mesenteric defect (Fig. 3) and meconium-stained ascites related to a bowel perforation (Fig. 4). However, the newborn grew well after surgical intervention. She was kept followed up at the pediatric outpatient clinic.

## Discussion

Volvulus is the strangulation or malrotation of the intestines, including both small and large intestines. It is not rare for young children, with symptoms of painful abdominal distention, constipation, and acute abdomen. Emergent surgical intervention is mandatory to improve health and even save the life of a patient [2]. Although it is relatively rare, volvulus might be seen during the intrauterine life. This incidence is around 1/6,000 of live births [3].

Small bowel obstructions are rarely detected before the second trimester of pregnancy. During the third trimester, bowel loops become more dilated with more vigorous peristalsis. However, intestinal echo images are not easily detected during intrauterine life. Investigation for such abnormality is needed with the sign of an enhanced echogenic intestine. Duodenal atresia or ileal atresia is highly suspected when small bowel dilatation is found. Dilatation of the large bowel might be the result of megacolon. Although volvulus of the midgut is rare, it should be highly suspected given specific sonar features, such as whirlpool sign, coffee bean sign [4], or abruptly and markedly dilated bowel [5]. It is challenging to diagnose intrauterine volvulus. Not all reported cases have typical ultrasound features or malrotation [5]. Most of these cases were suspected based on the antenatal findings of markedly dilated intestinal loops [5]. The definitive diagnosis is made only after the neonate’s surgical intervention [1]. Likewise, in our case, we found a similar feature of marked intestinal dilatation, which gave the impression of bowel obstruction and the suspicion of intrauterine volvulus. Fetal MRI is likely helpful in precisely locating the site of obstruction and in differential diagnosis [1]. Although similar images can be obtained by MRI examination [1,4], the downside is its higher cost, longer examination time, and the technical expertise required, making this modality impractical [5]. All the specific sonar findings indicated bowel obstruction, which was an emergent condition of the fetus.

In the event of malrotation, the bowel loop of volvulus would be twisted around the fetal mesenteric artery or its branches, resulting in intestinal obstruction and vascular compromise. All of these can cause in the fetus bowel infarction, necrotic bowel perforation [6], with the development of hemorrhagic ascites, anemia [6], and even death if untreated [5,7]. Clinical consequences may include preterm labor, premature rupture of membranes, polyhydramnios, diminishing fetal movements, loss of fetal heartbeat variability, and non-reactive non-stress test [5,7]. Preterm labor or delivery is known to be caused by the activation of adrenal and hypothalamic hormones, secondary to an acute fetal distress [5,8,9]. In the presence of any of the above warning signs, a prompt delivery is mandatory. Meanwhile, the neonate needs to undergo emergent exploratory laparotomy to relieve timely the intestinal obstruction. Delays in delivery or neonatal exploratory laparotomy may cause intrauterine fetal death or neonatal mortality [2].

In our case, we found a markedly dilated bowel. At first, based on the sonar findings, intrauterine volvulus was highly suspected before the termination of pregnancy. Definitive diagnosis was explored when the neonate underwent exploratory laparotomy after birth. The volvulus was found to be the result from the intestine penetrating through a mesenteric defect, in addition to the meconium ascites related to an intestinal perforation. The newborn did well after bowel operation and in subsequent follow-ups.

Early detection of intrauterine volvulus is key to reducing its morbidity and mortality [6]. In isolated abnormality cases, prognosis is good with >95% chance of survival. The outcome depends on the gestational stage at the time of diagnosis [10] and the amount of residual bowel. Short bowel syndrome should be also watched out after operation.

## Conclusions

Although the American College of Obstetrician and Gynecologist and the American College of Radiology do not recommend regular procedure of fetal sonography during the third trimester, first, the procedure should be done carefully especially in the presence of obstetrical complications, with emphasis on imaging the gastrointestinal tract. Second, close monitoring of the fetal condition should be carried out in the case of abnormally distended bowel. Meanwhile, a possible increase in the frequency of anomaly ultrasound screening is a more feasible modality based on the cost factor. Third, a prompt delivery should be done when the fetal condition deteriorates, such as diminishing fetal movements or abnormal patterns of fetal heartbeat. Fourth, an expert neonatologist is required for special newborn care before and after operation. Fifth, a pediatric surgeon should be arranged for urgent operation to relieve the intestinal obstruction. Sixth, a special dietician is needed for nutritional care after operation.
